# Associations between Extreme Precipitation and Gastrointestinal-Related Hospital Admissions in Chennai, India

**DOI:** 10.1289/ehp.1306807

**Published:** 2013-12-17

**Authors:** Kathleen F. Bush, Marie S. O’Neill, Shi Li, Bhramar Mukherjee, Howard Hu, Santu Ghosh, Kalpana Balakrishnan

**Affiliations:** 1 Department of Environmental Health Sciences,; 2 Department of Epidemiology,; 3 Risk Science Center, and; 4 Department of Biostatistics, School of Public Health, University of Michigan, Ann Arbor, Michigan, USA; 5 Division of Global Health,; 6 Division of Epidemiology, and; 7 Division of Occupational & Environmental Health, Dalla Lana School of Public Health, University of Toronto, Toronto, Ontario, Canada; 8 Department of Environmental Health Engineering, Sri Ramachandra University, Chennai, India

## Abstract

Background: Understanding the potential links between extreme weather events and human health in India is important in the context of vulnerability and adaptation to climate change. Research exploring such linkages in India is sparse.

Objectives: We evaluated the association between extreme precipitation and gastrointestinal (GI) illness-related hospital admissions in Chennai, India, from 2004 to 2007.

Methods: Daily hospital admissions were extracted from two government hospitals in Chennai, India, and meteorological data were retrieved from the Chennai International Airport. We evaluated the association between extreme precipitation (≥ 90th percentile) and hospital admissions using generalized additive models. Both single-day and distributed lag models were explored over a 15-day period, controlling for apparent temperature, day of week, and long-term time trends. We used a stratified analysis to explore the association across age and season.

Results: Extreme precipitation was consistently associated with GI-related hospital admissions. The cumulative summary of risk ratios estimated for a 15-day period corresponding to an extreme event (relative to no precipitation) was 1.60 (95% CI: 1.29, 1.98) among all ages, 2.72 (95% CI: 1.25, 5.92) among the young (≤ 5 years of age), and 1.62 (95% CI: 0.97, 2.70) among the old (≥ 65 years of age). The association was stronger during the pre-monsoon season (March–May), with a cumulative risk ratio of 6.50 (95% CI: 2.22, 19.04) for all ages combined compared with other seasons.

Conclusions: Hospital admissions related to GI illness were positively associated with extreme precipitation in Chennai, India, with positive cumulative risk ratios for a 15-day period following an extreme event in all age groups. Projected changes in precipitation and extreme weather events suggest that climate change will have important implications for human health in India, where health disparities already exist.

Citation: Bush KF, O’Neill MS, Li S, Mukherjee B, Hu H, Ghosh S, Balakrishnan K. 2014. Associations between extreme precipitation and gastrointestinal-related hospital admissions in Chennai, India. Environ Health Perspect 122:249–254; http://dx.doi.org/10.1289/ehp.1306807

## Introduction

Global climate change is expected to increase the frequency, intensity, and duration of extreme weather events, with potential adverse effects on human health. High-risk areas include those already experiencing a scarcity of resources, environmental degradation, high rates of infectious disease, weak infrastructure, and overpopulation (Patz et al. 2005). Vulnerable populations include the elderly, children, urban populations, and the poor (Ebi and Paulson 2010; Gangarosa et al. 1992; O’Neill and Ebi 2009; Trinh and Prabhakar 2007). Understanding the relationship between climate variability and human health in India is important as India integrates existing public health programs with climate change adaptation strategies and early warning systems (Bush et al. 2011).

Diarrheal disease remains among the top five causes of death in low- and middle-income countries, particularly among children under 5 years of age (Boschi-Pinto et al. 2008). However, research linking weather variability to diarrheal disease in India is sparse. Evidence from elsewhere in the world suggests that waterborne disease outbreaks are preceded by extreme precipitation events (Curriero et al. 2001) and that the seasonal contamination of surface water may explain some of the variability in the occurrence of many waterborne diseases (Patz et al. 2008). Outbreaks of Cholera were linked to extreme precipitation and temperature in the Lake Victoria Basin (Olago et al. 2007), Bangladesh (Pascual et al. 2000, 2008), and Peru (Checkley et al. 2000). Further evidence suggests that seasonal changes in temperature and precipitation affect the incidence of cryptosporidiosis around the world (Jagai et al. 2009). High levels of water volume were associated with infectious gastrointestinal (GI) illness in northern Canada (Harper et al. 2011) as well as cases of rotavirus infection in Bangladesh (Hashizume et al. 2007). In Taiwan, extreme precipitation was linked to waterborne infections (Chen et al. 2012). Thus, evaluating the association between extreme precipitation and GI illness in Chennai, India, contributes valuable site-specific information to a growing set of literature on the topic. The primary goal of the present study was to evaluate the association between extreme precipitation and GI-related hospital admissions over a 15-day period using a distributed lag framework.

## Data and Methods

**Figure 1:**
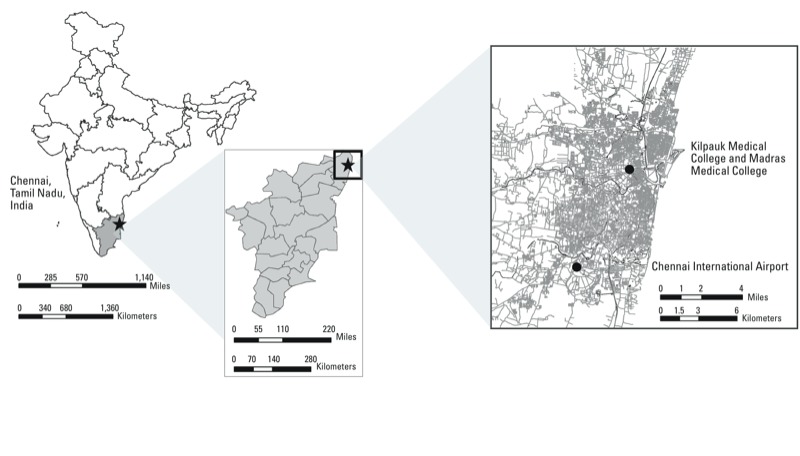
Location of Chennai, India, depicting the location of Chennai within the state of Tamil Nadu as well as the locations of Kilpauk Medical College, Madras Medical College, and Chennai International Airport.

*Study location*. The study was conducted in Chennai, the capital city of India’s southern state, Tamil Nadu (Figure 1). Chennai has an estimated population of 4.68 million people and is one of the most densely populated cities in the world. Approximately 78% of Chennai’s population has access to tap water from a treated source and 58% to a piped sewage connection (Government of Tamil Nadu 2011). Nearly 10% of Chennai’s population lives in disadvantaged, slum-like settings where access to safe drinking water is severely limited (Chandramouli 2003; McKenzie and Ray 2009).

*Hospital admission data*. Daily hospital admission data for the period of 2004 to 2007 were collected from two government hospitals in Chennai (Madras Medical College and Kilpauk Medical College) after obtaining relevant approval from the Directorate of Public Health, Government of Tamil Nadu. These two hospitals account for nearly 50% of available beds in government facilities in Chennai. A third government facility in Chennai, Stanley Medical Hospital, provides another 25%, and the last 25% is provided by several smaller facilities. In general, Indian government hospitals serve lower socioeconomic patients, whereas the majority of middle-class and high-income patients are served by private medical facilities. Thus, although these two government hospitals represent only a fraction of Chennai’s overall population, they represent a strong majority of the low-socioeconomic population. These data were cleaned and organized in support of previously published analyses (Balakrishnan et al. 2011).

Hospital admissions were defined as GI-related if the primary, secondary, or tertiary *International Statistical Classification of Diseases and Related Health Problems, Tenth Revision* [ICD-10; World Health Organization (WHO) 1994] code was listed as intestinal infectious disease (codes A00–A09), helminthiases (codes B65–B83), or GI-related symptoms (codes R11-nausea and vomiting, R50-fever, R51-headache). Cases were selected by matching ICD-10 codes to *International Classification of Diseases, 1975 Revision* (ICD-9) (WHO 1977) codes used in previous research (Morris et al. 1996; Schwartz et al. 2000). Data from the two hospitals were combined and collapsed into daily hospitalization counts of GI illnesses. Admissions lacking an ICD-10 code were categorized as unclassified.

*Meteorological data*. Daily meteorological data, monitored at the Chennai International Airport (Figure 1) and available from the National Oceanic and Atmospheric Administration’s National Climatic Data Center (2011) Global Surface Summary of the Day were also collected for the period 2004–2007. Parameters extracted included precipitation, temperature, dew point, and relative humidity.

For our analysis, daily precipitation was categorized using the overall distribution during the 2004–2007 study period to assign cut points. Precipitation categories were defined as 0 mm (reference category); > 0 mm, but < 90th percentile (approximately 12 mm, or 0.5 inches); and ≥ 90th percentile. The 90th percentile was chosen as the cutoff based on previous research stating that a majority of waterborne outbreaks were preceded by extreme precipitation, above the 90th percentile (Curriero et al. 2001; Rose et al. 2000). This analysis focuses on the effects of extreme precipitation relative to zero precipitation.

*Statistical analysis*. We hypothesized that extreme precipitation (≥ 90th percentile) would be associated with an increased risk of GI-related hospital admissions but not all-cause hospital admissions. Evaluating the association between extreme precipitation and all-cause admissions served as a negative control, providing evidence that any observed association between extreme precipitation and GI illness was not simply an artifact of the time–series data.

Generalized additive models were fit with daily counts of hospital admissions as the dependent variable and categorical daily precipitation as the independent variable, adjusted for potential confounders (Hastie and Tibshirani 1986, 1990). In order to control for long-term time trends in hospital admissions, a nonlinear smoothing term for time (i.e., a penalized spline) was included. The smoothing parameters were chosen to minimize the generalized cross validation (GCV) score in the generalized additive model (Hastie and Tibshirani 1986, 1990). An over-dispersion parameter was included to account for instances where the sample variance differed from the sample mean (McCullagh and Nelder 1989). Dean’s test was used to evaluate overdispersion (Dean 1992).

Potential confounders. Daily average apparent temperature (AT), defined as 2.653 + (0.994 × *T_a_*) + (0.0153 × *T_d_*^2^), where *T_a_* is air temperature (°C) and *T_d_* is dew point temperature (°C) (Kalkstein and Valimont 1986; Steadman 1979), was included as a potential confounder. AT was used because it represents the combined effects of temperature and humidity, which have been linked to the replication, persistence, and transmission of pathogens in the environment (Checkley et al. 2000; Fleury et al. 2006; Naumova et al. 2007; Singh et al. 2001) and the health of vulnerable populations (Kovats and Akhtar 2008; Trinh and Prabhakar 2007). All models included average daily apparent temperature on the day of hospitalization as a continuous variable.

An indicator variable representing the day of week (DOW) of hospitalization was also included as a potential confounder. Because the very young and the very old are often at increased risk of hospitalization, we performed separate analyses stratified by age. “Young” was defined as ≤ 5 years of age, “old” was defined as ≥ 65 years of age, and “intermediate” as 6–64 years of age. Models were not adjusted for holidays.

Lags. Based on previous reports, GI-related hospital admissions were expected to peak several days after the occurrence of an extreme precipitation event because of delayed environmental transport of pathogens and delayed onset of clinical symptoms. Previous studies have reported a delayed onset of symptoms and subsequent hospitalization following extreme precipitation (Aramini et al. 2000; Curriero et al. 2001; Egorov et al. 2003; Schwartz et al. 2000). Incubation periods of waterborne pathogens can range from 1 day (e.g., for *Shigella, Salmonella*, and *Rotavirus*) to up to 2 weeks (e.g., for *Cryptosporidium* and *Escherichia coli*) (Haley et al. 2009; Jagai et al. 2009). To account for this variability, the association was explored across a 15-day lag.

Initial exploratory analysis comprised fitting generalized additive models with 15 separate single-day lags before the day of hospital admission (model 1):

log[*E*(*HA_t_*)] *=* β_0_ *+* β_1_*PRCP_t–q_ +* β_2_*AT_t_ +* β_3_*DOW_t_ + s*_1_(*time*), [1]

where *HA* is the number of hospital admissions, *PRCP* is the daily precipitation variable, *t* represents the day of hospitalization, *q* denotes single-day lags 1–15 days before the day of hospital admission (*q = 1, 2, …, 15*), *AT* is daily average apparent temperature, *DOW* is day of week, and *s_1_*(*time*) is a penalized spline using calendar time with smoothing parameters chosen to minimize the generalized cross-validation score.

The main analysis included a distributed lag model (Schwartz 2000; Zanobetti et al. 2000) to evaluate the cumulative effect over a 15-day period following an extreme precipitation event (Gasparrini et al. 2010). Distributed lag models, common in air pollution studies (Schwartz 2000; Zanobetti et al. 2000), provide a systematic way to investigate the distribution of effects over time. We constrained model coefficients using the lag number to fit a polynomial function (Schwartz 2000; Zanobetti et al. 2000) to reduce collinearity resulting from correlated levels of precipitation on days that are close together in time. This approach allows the cumulative effect of precipitation to be modeled over the entire lag period, simultaneously estimating the nonlinear and delayed effects (model 2):

log[*E*(*HA_t_*)] *=* β_0_ *+* Σ^^15^^*_q =_*_1_ α*_q_PRCP_t–q_ +* β_2_*AT_t_ +* β_3_*DOW_t_ + s*_2_(*time*), [2]

where α*_q_* is the effect of extreme precipitation *q* days before the day of hospitalization and *s*_2_(*time*) is a penalized spline using calendar time with smoothing parameters chosen to minimize the generalized cross validation score. The cumulative summary of risk ratio estimates corresponding to extreme precipitation is given by Σ^^15^^*_q =_*_1_ α*_q._*

Seasonal analysis. The Indian monsoon season is characterized by extreme precipitation that contributes to > 85% of India’s annual rainfall (Vialard et al. 2011). A stratified analysis explored the association across seasons defined according to the Indian Meteorological Department (2011) and Vialard et al. (2011) as: winter (January–February), pre-monsoon (March–May), early monsoon (June–September), and late monsoon (October–December). In considering only one season, for example winter, a discontinuous time series associated with the outcome variable would normally be introduced in the transition from one winter to the next. Whereas this naïve method would string the four winters together and ignore that discontinuity in the temporal profile, we adopted a two-stage approach that first estimates the spline term based on the entire time series using all days and a simple unadjusted Poisson regression model (model 3) and then incorporates the spline estimates as an offset in the full regression model (model 4):

log[*E*(*HA_t_*)] *= s*_3_(*time*), [3]

log[*E*(*HA_t_*)] *=* β*_0_ +* Σ^^15^^*_q =_*_1_ α*_q_PRCP_t–q_ +* β_2_*AT_t_ +* β_3_*DOW_t_ + offset_t_*, [4]

where *offset* represents the estimated spline terms *s*_3_(*time*) from the full time series evaluated at day *t*.

Sensitivity analysis. Because the annual precipitation distribution is heavily influenced by the monsoon, a sensitivity analysis was conducted to compare the effect of extreme precipitation between the predominantly wet season and the rest of the year: late monsoon (October–December) compared with dry (January–September). A sensitivity analysis was also run excluding 2004 data from all analyses in order to confirm that missing data early in the study period did not bias the results.

For all models, cumulative risk ratio estimates were calculated corresponding to extreme daily precipitation (≥ 90th percentile), where zero precipitation was the reference category. Estimates from the distributed lag models represent the cumulative summary of risk ratio estimates of a hospital admission (for GI-related, all-cause, or unclassified cases) during 15-day periods corresponding to an extreme precipitation event (a day with precipitation ≥ 90th percentile) relative to the cumulative risk during 15-day periods following days with no precipitation. The level of significance for all statistical tests was set to 0.05. Analyses were run using SAS (version 9.2; SAS Institute Inc., Cary, NC, USA) GAM package (Hastie and Tibshirani 1986, 1990) and R (R Foundation for Statistical Computing, Vienna, Austria) DLNM package (Gasparrini et al. 2010).

## Results

**Figure 2:**
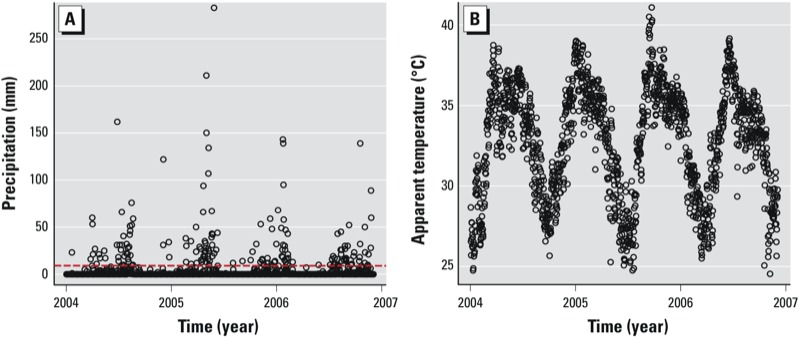
Mean daily precipitation (*A*) and mean daily apparent temperature (*B*) in Chennai, India, from 2004 to 2007. The 90th percentile is indicated as a red dashed line in *A*.

*Descriptive analysis*. Daily precipitation totals during the study period ranged from 0 to 283 mm with a daily mean of 4.48 mm (Table 1, Figure 2A). The range in daily mean precipitation varied from 3.45 mm in 2007 to 6.40 mm in 2005; there were several more days with precipitation totals > 100 mm in 2005 compared with other years. Seasonal precipitation varied with the onset of the monsoon; daily mean precipitation varied from 0.17 mm in winter to 10.73 mm in late monsoon. Precipitation showed a skewed distribution; out of a total 1,461 days, 991 days (68%) had 0 mm precipitation and 424 days (29%) had greater than 0 mm. Precipitation data were missing on 46 days (3%). The 90th percentile of precipitation used as the cut point in the analysis was 11.94 mm. The number of extreme events also varied with season with 10 events during winter, 32 events during pre-monsoon, 70 events during early monsoon, and 32 events during late monsoon. Daily average apparent temperature was consistently near 33°C (91°F) across years (Figure 2B), whereas apparent temperature showed slight variation across seasons: 28°C during winter and 35°C during pre- and early-monsoon.

**Table 1:** Daily average meteorological conditions categorized by year and by season in Chennai, India, 2004–2007 [mean; median (range)] and number of extreme events within each category.

Variable	Precipitation (mm)	Apparent temperature (°C)	Extreme events (*n*)
By year
2004	4.05; 0 (0–162)	33; 34 (25–39)	34
2005	6.40; 0 (0–283)	33; 34 (25–39)	44
2006	4.03; 0 (0–143)	33; 34 (25–41)	34
2007	3.45; 0 (0–139)	32; 33 (25–39)	32
By season
Winter (January–February)	0.17; 0 (0–23)	28; 28 (25–33)	10
Pre-monsoon (March–May)	1.35; 0 (0–123)	35; 35 (29–41)	32
Early monsoon (June–September)	4.23; 0 (0–162)	35; 35 (29–39)	70
Late monsoon (October–December)	10.73; 0 (0–283)	31; 30 (25–36)	32
Dry (January–September)	2.38; 0 (0–162)	34; 35 (25–41)	112
Entire period (2004–2007)^*a*^	4.48; 0 (0–283)	33; 33 (25–41)	144
^***a***^The 90th percentile for the entire study period (11.94 mm) was used to define extreme precipitation.			

GI-related hospital admissions accounted for approximately 4% of all hospital admissions (Table 2). Although unclassified admissions also accounted for approximately 4% of all hospital admissions, they ranged from 1% during 2004–2006 to 11% in 2007. This spike in unclassified admissions could not be systematically explained. The number of all-cause hospital admissions varied from 57,237 in winter to 107,809 in early monsoon; GI-related admissions varied from 2,344 in winter to 4,893 in early monsoon; unclassified hospital admissions ranged from 1,090 in winter to 5,265 in late monsoon (Table 2).

**Table 2:** Daily hospital admissions [mean (young, ≤ 5 years; old, ≥ 65 years)] by year, season, age, and cause from two government hospitals in Chennai, India, 2004–2007.

Variable	All-cause	GI-related^*a*^	Unclassified
By year
2004^*b*^	46,981 (1,788; 4,295)	2,639 (153; 248)	440 (11; 38)
2005	76,170 (3,570; 7,156)	4,321 (195; 403)	1,094 (30; 38)
2006	117,508 (10,131; 9,541)	4,692 (130; 482)	1,282 (41; 53)
2007	95,065 (9,537; 7,731)	3,071 (73; 345)	10,923 (102; 1,143)
By season
Winter (January–February)	57,237 (3,699; 5,105)	2,344 (69; 241)	1,090 (25; 63)
Pre-monsoon (March–May)	84,444 (5,440; 7,153)	3,550 (117; 353)	3,519 (45; 324)
Early monsoon (June–September)	107,809 (8,616; 8,979)	4,893 (180; 491)	3,865 (81; 273)
Late monsoon (October–December)	86,234 (7,301; 7,486)	3,936 (185; 393)	5,265 (33; 612)
Entire period (2004–2007)	335,724 (25,026; 28,723)	14,723 (551; 1,478)	13,739 (184; 1,272)
^***a***^Cases were defined as GI-related if the primary, secondary, or tertiary ICD-10 code was listed as intestinal infectious disease (codes A00–A09), helminthiases (codes B65–B83), or GI-related symptoms (codes R11-nausea and vomiting, R50-fever, R51-headache). ^***b***^2004 data from Kilpauk Medical College were limited to May–December.			

*Main effect analysis.* Exploratory analysis using single-day lag models indicated that extreme precipitation was associated with GI illness at later lags (lags 6, 8, 10, 11, 14, and 15 indicated a positive association) for the overall population (see Supplemental Material, Table S1). For example, GI-related hospital admissions had a risk ratio of 1.10 (95% CI: 1.02, 1.17) at lag 10 and 1.14 (95% CI: 1.07, 1.22) at lag 15. Unexpectedly, extreme precipitation showed a protective effect for unclassified hospital admissions at lags 7 through 15.

In the distributed lag model, extreme precipitation was significantly associated with GI-related hospital admissions with a cumulative risk ratio equal to 1.60 (95% CI: 1.29, 1.98) controlling for AT, DOW, and long-term time trends (Table 3).

**Table 3:** Cumulative risk ratio effects of hospitalization associated with extreme precipitation (≥ 90th percentile) by cause of admission and age category based on the 15-day distributed lag model.

Age category	Cause of admission	Cumulative RR (95% CI)
All ages	All-cause	1.01 (0.89, 1.16)
	GI-related^*a*^	1.60 (1.29, 1.98)
	Unclassified	0.33 (0.19, 0.58)
Young (≤ 5 years)	All-cause	1.04 (0.82, 1.32)
	GI-related	2.72 (1.25, 5.92)
	Unclassified	0.86 (0.24, 3.08)
Old (≥ 65 years)	All-cause	0.99 (0.82, 1.19)
	GI-related	1.62 (0.97, 2.70)
	Unclassified	0.11 (0.03, 0.37)
Intermediate (6–64 years)	All-cause	1.05 (0.92, 1.21)
	GI-related	1.61 (1.27, 2.03)
	Unclassified	0.17 (0.10, 0.32)
RR, risk ratio. All models control for daily average apparent temperature on the day of hospitalization, day of week, and time. ^***a***^Cases were defined as GI-related if the primary, secondary, or tertiary ICD-10 code was listed as intestinal infectious disease (codes A00–A09), helminthiases (codes B65–B83), or GI-related symptoms (codes R11-nausea and vomiting, R50-fever, R51-headache).		

Among the young, the cumulative risk ratio of GI-related hospital admissions was 2.72 for a 15-day period following an extreme event compared with a 15-day period following days with no precipitation (95% CI: 1.25, 5.92). Among the old, the association was also positive, but not statistically significant with a cumulative risk ratio of 1.62 (95% CI: 0.97, 2.70). As expected, results for the intermediate age group were consistent with the overall population: there was a positive association for GI-related admissions with a cumulative risk ratio of 1.61 for a 15-day period following an extreme event (95% CI: 1.27, 2.03) and no association for all-cause admission. Unclassified admissions revealed a negative association among the overall, old, and intermediate age groups.

*Seasonal analysis*. Using the two-stage technique within the distributed lag framework, extreme precipitation was associated with both all-cause and GI-related hospital admissions during the pre-monsoon season with a cumulative risk ratio of 4.61 (95% CI: 2.57, 8.26) and 6.50 (2.22, 19.04), respectively (Table 4). Models stratified by both age and season did not always converge because of low counts of hospital admissions and too few extreme precipitation events (results not shown).

**Table 4:** Comparing cumulative risk ratio effects of hospitalization associated with extreme precipitation (≥ 90th percentile) across seasons by cause of admission for all ages based on the 15-day distributed lag model.

Cause of admission	Pre-monsoon (March–May)	Early monsoon (June–September)	Late monsoon (October–December)	Dry (January–September)
All-cause	4.61 (2.57, 8.26)	1.17 (0.73, 1.87)	0.79 (0.69, 0.92)	1.70 (1.24, 2.33)
GI-related^*a*^	6.50 (2.22, 19.04)	0.63 (0.28, 1.45)	0.95 (0.75, 1.20)	1.88 (1.06, 3.33)
Unclassified	3.15 (0.29, 34.23)	1.86 (0.35, 9.79)	1.00 (0.45, 2.19)	1.68 (0.41, 6.95)
All models control for daily average apparent temperature on the day of hospitalization, day of week and time. Season-specific estimates are reported for all ages due to a lack of model convergence when stratified by both age and season. No results are presented for Winter (January–February) because of a lack of model convergence. ^***a***^Cases were defined as GI-related if the primary, secondary, or tertiary ICD-10 code was listed as intestinal infectious disease (codes A00–A09), helminthiases (codes B65–B83), or GI-related symptoms (codes R11-nausea and vomiting, R50-fever, R51-headache).				

Results from the seasonal sensitivity analysis were largely consistent with the overall analysis (Table 4). The dry season, defined as January–September, followed a similar pattern as the pre-monsoon season, defined as March–May, with positive associations for both all-cause and GI-related hospital admissions. Cumulative risk ratios during the dry season were equal to 1.70 (95% CI: 1.24, 2.33) and 1.88 (95% CI: 1.06, 3.33) for all-cause and GI-related hospital admissions, respectively.

Discussion

GI-related hospital admissions in Chennai were consistently associated with extreme precipitation (≥ 90th percentile) over a 15-day lag. A study based in northern Canada reported similar results: high water volume was associated with a 1.34-times increase in the number of GI-related clinic visits over a 2-week lag (*p* < 0.05) (Harper et al. 2011). Another study reported that rainfall events above the 93rd percentile were associated with a 2.28-times (95% CI: 1.22, 4.23) increase in the risk of waterborne outbreaks (Thomas et al. 2006). Previous work also explored the effect of individual infectious agents, suggesting that heavy precipitation was associated with a 2.45-times (95% CI: 1.59, 3.78) increase in *Enterovirus* and that torrential precipitation was associated with a 2.85-times (95% CI: 2.06, 3.95) increase in Bacillary dysentery (Chen et al. 2012). Another study concluded that an interquartile range increase in drinking-water turbidity (likely a result of extreme precipitation) was positively associated with the increased risk of hospital admissions among children 0–15 years of age at lags 8 and 13 (Schwartz et al. 1997); these findings are consistent with our results reporting a significant association between extreme precipitation and GI-related hospital admissions among the young over a 15-day lag. Although previous studies have incorporated a lagged effect, they have not presented results from a distributed lag model as we do here.

The seasonal analysis revealed a significant association between extreme precipitation and the risk of GI-related hospital admissions during the drier, pre-monsoon season. Although previous studies have not evaluated the impact of extreme events during relatively dry periods, they do suggest that admission rates are elevated during both high and low rainfall extremes in Bangladesh (Hashizume 2007); in England and Wales, waterborne disease outbreaks were preceded by both high and low rainfall (Nichols et al. 2009). Although several studies have characterized the Indian monsoon season, few have examined the impact of heavy precipitation on the burden of waterborne disease in this region. Recent findings report an increased frequency of heavy rain events, but with a decreased number of rainy days and a decrease in total precipitation (Kumar and Jain 2011). In addition, the number of severe cyclonic storms and the amount of rainfall off the Indian Coast has increased, with an observed decrease in precipitation during the summer monsoon and an increasing trend in precipitation during both the pre-monsoon and post-monsoon (Dash et al. 2007). In some cases, severe outbreaks of waterborne disease have been directly associated with flooding (Sur et al. 2000).

The association between extreme precipitation and GI-related hospital admissions could have important implications for public health and water-resource professionals in low- and middle-income countries that have a high burden of GI illness. Sanitation, access to treated tap water, and piped sewage connections are necessary to reducing the overall risk of GI-related hospital admissions. These findings suggest that heightened vigilance after an extreme precipitation event, raising awareness of the potential link between extreme precipitation and GI-related hospital admissions, and the creation of early-warning systems based on weather prediction models could form an interim solution.

In a majority of low- and middle-income countries, meteorological data are not easily linked to health data. Although ecological studies using time–series analysis can serve as a cost-effective design for examining associations, the logistical challenges of data collection often preclude development of an analytical framework. We were able to use a high-quality data set, leveraging data collected for a study investigating air pollution and health effects in Chennai (Balakrishnan et al. 2011). Studying health impacts across seasons and during extreme weather events can aid in preparation for a future in which such extremes are expected to become more common (Cooney 2011).

The primary limitation of the present study is that GI illness remains highly underreported so only a subset of cases are identified (Charron et al. 2004; Ford 1999). A second limitation is related to data quality. It is clear that 2004 had fewer daily hospital admissions than other years because data from Kilpauk Medical College were limited to May–December. To confirm that this trend did not bias the results, models were rerun excluding 2004 data. The cumulative risk ratios corresponding to GI-related hospital admissions among the young were very consistent, 2.71 (95% CI: 1.27, 5.78); however, the cumulative risk ratios corresponding to GI-related hospital admissions among the general population and among the old were no longer significant, with estimates of 1.09 (95% CI: 0.79, 1.51) and 1.37 (95% CI: 0.76, 2.49), respectively (see Supplemental Material, Table S2). Acknowledging this limitation, we included 2004 data in our primary analysis in order to maximize our sample size. Nevertheless, our use of a unique 4-year time series of cause-specific hospital admission data and meteorological data for one of the largest cities in India is an important contribution to the climate-health literature; future work should focus on additional climate-sensitive health outcomes in low- and middle-income countries.

Changing water consumption patterns and increased pressure on water systems from growing urban populations and expanding agriculture will add additional pressure to an already overburdened water system. These various factors related to water quality and quantity could create high-risk scenarios for water contamination during heavy rain events. Thus, future work should evaluate how changing land use patterns and population density influence the risk of waterborne disease. In light of the multiple environmental threats that India may face in the years ahead (Rao 2010), the impacts of climate change must be evaluated in the context of other global environmental factors. Environmental parameters measured by remote satellite imaging and subsequent indicators have the potential to not only provide global coverage of changing environmental conditions, but to also predict future risks and inform adaptation strategies (Ford et al. 2009).

## Conclusions

We explored the association between extreme precipitation (≥ 90th percentile) and GI-related hospital admissions in Chennai, using a 4-year time–series data set. The cumulative risk ratio for GI-related hospital admissions following extreme precipitation events was higher among the young (≤ 5 years of age) compared with the overall population. These results, in combination with projected changes in precipitation, suggest that climate change will have important implications for human health in India where global health disparities and challenges in water resource management already exist.

## Supplemental Material

(61 KB) PDF
