# Supplementary material for: Associations between Extreme Precipitation and Gastrointestinal-Related Hospital Admissions in Chennai, India
Source: Environ Health Perspect. 2013 Dec 17;122(3):249–54. doi: 10.1289/ehp.1306807 (PMC3948034; doi:10.1289/ehp.1306807)
Supplement: (61 KB) PDF [file ehp.1306807.s001.pdf]

*Environmental Health Perspectives* doi:10.1289/ehp.1306807

# Supplemental Material

# Associations between Extreme Precipitation and Gastrointestinal-Related Hospital Admissions in Chennai, India

Kathleen F. Bush, Marie S. O'Neill, Shi Li, Bhramar Mukherjee, Howard Hu, Santu Ghosh, and Kalpana Balakrishnan

**Supplemental Material, Table S1.** Risk ratios (95% CI) corresponding to hospitalization associated with precipitation (≥ 90th percentile) by cause of admission for the single-day lag model.

| Lag | All-cause | GI-related | Unclassified |
|---|---|---|---|
| 0 | 0.93 (0.89, 0.97) | 0.93 (0.85, 1.01) | 1.04 (0.89, 1.20) |
| 1 | 0.95 (0.90, 0.99) | 0.91 (0.83, 0.99) | 0.97 (0.81, 1.12) |
| 2 | 0.96 (0.92, 1.01) | 1.00 (0.92, 1.07) | 0.93 (0.77, 1.09) |
| 3 | 0.98 (0.93, 1.02) | 1.01 (0.93, 1.09) | 0.97 (0.82, 1.13) |
| 4 | 0.97 (0.93, 1.01) | 1.02 (0.95, 1.10) | 0.89 (0.73, 1.05) |
| 5 | 0.97 (0.93, 1.01) | 1.02 (0.94, 1.10) | 0.88 (0.72, 1.04) |
| 6 | 0.96 (0.92, 1.00) | 1.10 (1.03, 1.18) | 0.84 (0.68, 1.00) |
| 7 | 0.95 (0.91, 1.00) | 1.04 (0.96, 1.12) | 0.78 (0.62, 0.95) |
| 8 | 0.96 (0.92, 1.00) | 1.09 (1.01, 1.16) | 0.77 (0.61, 0.93) |
| 9 | 0.96 (0.92, 1.00) | 1.05 (0.98, 1.13) | 0.76 (0.60, 0.93) |
| 10 | 0.97 (0.92, 1.01) | 1.09 (1.02, 1.17) | 0.70 (0.53, 0.86) |
| 11 | 0.96 (0.92, 1.01) | 1.10 (1.02, 1.17) | 0.59 (0.42, 0.76) |
| 12 | 0.97 (0.93, 1.02) | 1.06 (0.98, 1.14) | 0.61 (0.44, 0.78) |
| 13 | 0.98 (0.94, 1.03) | 1.07 (0.99, 1.14) | 0.67 (0.51, 0.84) |
| 14 | 1.00 (0.96, 1.04) | 1.08 (1.01, 1.16) | 0.75 (0.58, 0.92) |
| 15 | 1.02 (0.98, 1.06) | 1.14 (1.07, 1.22) | 0.75 (0.58, 0.92) |

All models control for daily average apparent temperature, day of week and time. Cases were defined as GI-related if the primary, secondary, or tertiary ICD-10 code was listed as intestinal infectious disease (A00-A09), helminthiases (B65-B83), or GI-related symptoms (R11-nausea and vomiting, R50-fever, R51-headache).

**Supplemental Material, Table S2.** Excluding 2004, risk ratio corresponding to hospitalization associated with precipitation (≥ 90th) percentile) by cause of admission and across age categories for distributed lag model.

| | Cause of admission | Cumulative RR (95% CI) |
|---|---|---|
| All ages | All-cause | 0.85 (0.72, 1.01) |
| | GI-related | 1.09 (0.79, 1.51) |
| | Unclassified | 0.04 (0.02, 0.11) |
| Young (≤ 5) | All-cause | 0.72 (0.52, 0.99) |
| | GI-related | 2.71 (1.27, 5.78) |
| | Unclassified | 0.19 (0.03, 1.49) |
| Old (≥ 65) | All-cause | 0.82 (0.63, 1.07) |
| | GI-related | 1.37 (0.76, 2.49) |
| | Unclassified | 0.07 (0.01, 0.36) |
| Intermediate (6-64) | All-cause | 0.88 (0.74, 1.05) |
| | GI-related | 1.14 (0.80, 1.61) |
| | Unclassified | 0.02 (0.01, 0.06) |

All models control for daily average apparent temperature on the day of hospitalization, day of week and time. Cases were defined as GI-related if the primary, secondary, or tertiary ICD-10 code was listed as intestinal infectious disease (A00-A09), helminthiases (B65-B83), or GI-related symptoms (R11-nausea and vomiting, R50-fever, R51-headache).
